# Understanding Disparities: Mental Health and Neurodevelopmental Challenges, Supports and Barriers for Immigrant Families in Canada

**DOI:** 10.3390/children12040468

**Published:** 2025-04-05

**Authors:** Rachel Germaine Cluett, Tasmia Hai

**Affiliations:** 1 Department of Psychology, Faculty of Arts, University of Manitoba, Winnipeg, MB R3T 2N2, Canada; cluettr@myumanitoba.ca; 2 Department of Educational and Counselling Psychology, McGill University, Montreal, QC H3A 1X1, Canada

**Keywords:** neurodevelopmental disorder, mental health, children, family, immigrant, support

## Abstract

Background: Neurodevelopmental disorders (NDDs) and mental health disorders (MH) present significant challenges to Canadian Children. While there is increased awareness, the NDD/MH service needs and barriers to service for immigrant children in Canada are unclear. Therefore, the present study explores NDD and MH problems and management among Canadian children compared to immigrant children. Method: An online survey was administered to eligible participants using AskingCanadians. A total of 682 parents (Mean age = 31.8, *SD* = 7.4), 41.3% of whom were immigrants, completed the survey. Participants were asked to complete questionnaires related to mental health in general, child MH and NDD service needs, social support and use and barriers to accessing services. Results: Results showed that immigrant participants reported significant underuse of child mental health services (1.5 times less use) despite a higher reported child need. Similarly, a higher frequency of children born to Canadian parents reported accessing NDD/MH assessment referrals compared to immigrant families. Parents of children referred for NDD/MH assessment also reported a higher prevalence of mood disorders and anxiety disorders. Furthermore, parents of children presenting with NDD/MH concerns overall reported a significantly higher impact of barriers to their child’s education compared to parents whose children did not present with NDD/MH concerns. This effect was driven by Canadian parents of children with NDD/MH reporting increased barriers. Conclusions: These findings highlight the importance of considering cultural background in clinical approaches to MDD/MH services. There is a need to increase awareness and reduce stigma regarding service access. Furthermore, the findings reiterate the ongoing challenges families of children with NDD/MH challenges face in accessing support.

## 1. Introduction

Children with prevalent mental health (MH) and neurodevelopmental disorders and their caregivers are at risk of increased psychological stress [1]. Neurodevelopmental disorders (NDDs) affect 14% of children worldwide, with an estimated prevalence of 9.5% for Canadian children [2]. Additionally, about 20% of Canadian youth report mental health challenges, including anxiety and mood disorders [3,4]. Children diagnosed with NDDs/MH report significant challenges in academic performance, social interactions and family dynamics [5,6]. Despite the increase in prevalence and awareness, there remain significant gaps in understanding if these rates represent all Canadian youth, including racialized children. Given that approximately 37% of children in Canada are a child of an immigrant, with this number expected to be closer to 49% by 2036 [7,8], it is important to understand the prevalence of NDDs/MH, service needs and barriers to service access in Canadian children of immigrants.

Newcomers and immigrant families often encounter challenges when migrating to a new country and adjusting to their new social and cultural community, including disparities in cultural norms, language barriers, employment hurdles, and the complex process of integrating into an unfamiliar environment [9,10,11]. These stressors are magnified for children and youth, who may exhibit social–emotional difficulties, such as low mood, impulsivity and distractibility, during this transitional period. There is a notable gap regarding the experiences of immigrant families and their children’s access to mental health and educational services. Children of immigrants show the lowest diagnostic prevalence estimates when compared to non-immigrant children, with 4.3% and 9.2% diagnostic rates, respectively [12]. However, it is unclear whether this rate represents the true prevalence rates of diagnosis or if it is due to the underutilization of mental health services.

Understanding the perspectives of immigrant families on NDD/MH service referrals, support and associated barriers is crucial for enhancing accessibility to interventions. The lack of available research on supports and challenges for families of children with NDDs is detrimental to providing accessible information to parents. Notably, immigrant families have reported feeling less knowledgeable and prepared about NDDs and how to address symptoms that their children are experiencing [13]. Additionally, these families also experience fewer external cues from social supports that their children may be displaying these symptoms and need extra support [14]. Teachers or other social supports who can identify problems early may be unable to effectively communicate concerns to families with a language or cultural barrier. Fewer external supports combined with lower feelings of competence and cultural belief around mental health problems can lead to unaddressed and unreported child mental health challenges.

### 1.1. NDD/MHs Prevalence in Immigrant Population

The prevalence of NDD/MHs among immigrant children is influenced by various factors, including socioeconomic status, cultural beliefs and access to healthcare. While there is no specific prevalence rate for NDDs among immigrant children in Canada, studies have shown that worldwide there is a prevalence of approximately 14% in children [8]. However, first-generation immigrants often have lower diagnosis rates compared to second- or third-generation children, which can be attributed to cultural or access barriers [15].

Several risk factors contribute to the prevalence and diagnosis of NDD/MHs among immigrant children: (1) Economic stressors: Children from low-income families, including immigrants, are more likely to experience stressors such as food insecurity, unstable housing and limited access to resources, which can exacerbate symptoms of NDD/MHs. (2) Cultural beliefs and language barriers: Cultural beliefs about child behaviour and mental health can influence the perception and reporting of NDD/MH symptoms. For instance, some cultures may view hyperactivity and impulsivity as normal behaviours, leading to underreporting of symptoms. Language barriers can also hinder communication with healthcare providers, making it difficult to convey symptoms accurately. (3) Access to healthcare: Immigrant families may face barriers in accessing healthcare services, including lack of familiarity with the healthcare system, difficulty navigating complex administrative tasks and limited financial resources. These barriers can delay diagnosis and treatment access, potentially worsening symptoms over time. (4) Parental stress: The stressors associated with migration, including adjusting to a new culture, language and social environment, can impact both the parents’ and children’s mental health. Elevated parental stress can contribute to discrepancies in NDD/MH symptom reports between parents and teachers, lowering diagnostic accuracy [16,17].

### 1.2. Barriers in Accessing Services

New immigrant families often lack knowledge about the Canadian healthcare system, which can lead to difficulty navigating and understanding available resources [18]. Additionally, complex administrative tasks such as documentation requirements and insurance coverage create more significant barriers to accessing these services [19] on top of already lengthy waitlists for medical care access. Without insurance, even publicly funded services remain prohibitive to individuals with limited financial resources, especially among families with limited access to employer-provided health insurance.

### 1.3. Present Study

With the present study, we seek to improve our understanding of the prevalence of child MH/NDD struggles in immigrant children, the use of mental health services and other support systems and school-based barriers from the perspective of immigrant parents. The immigrant population in this study is defined as parents who were born outside of Canada and have migrated to the country. We used an online survey to also better understand the impact on parental mental health, access to information about NDD/MHs and potential barriers faced when accessing services. These research questions contribute to a comprehensive understanding of the referral and support ecosystem for children with NDD/MHs, informing both clinical practice and policy development aimed at enhancing the well-being of affected children and their families.

### 1.4. Research Questions

The present study seeks to investigate the aspects of NDD/MH challenges, referral and support mechanisms from the perspective of parents.

(1) Do rates of mental health and neurodevelopmental challenges differ between children of immigrant background compared to children born to Canadian parents?
(2) What percentage of immigrant parents report that their child has been referred for an NDD/MH assessment compared to parents who are non-immigrants in a sample of community members?
(3) Are there differences in parent mental health reported for children who have been referred for an NDD/MH assessment?
(4) Where do Canadian parents seek out information about NDD/MH services?
(5) What are the different school-based barriers reported by parents of children referred for NDD/MHs assessment compared to children who are not referred?

## 2. Method

### 2.1. Study Design and Participants

An online survey was administered in English to participants via McGill University’s Qualtrics platform, encompassing primarily multiple-choice questions with a few short open-ended responses. The survey was distributed to parents across Canada through AskingCanadians (Available online: https://portal.askingcanadiansprojects.com/ [accessed on 5 March 2025]), a Canadian crowdsourcing data collection company. Participants with existing profiles were sent information about the study and recruited from a diverse national sample to ensure a broad representation of Canadian parents. Upon completing the survey, participants received compensation equivalent to $5.00 CAD. The study was approved by McGill University Research Ethics Board # 2 (REB # 23-09-053).

### 2.2. Inclusion Criteria

To be eligible for participation in the present study, individuals needed to be (a) at least 18 years old, (b) reside in Canada, (c) be caregivers to a child aged between 5 and 17 years and (d) able to read, write and understand English and have access to an electronic device with internet capability (e.g., smartphone, tablet, computer).

### 2.3. Measures

This online survey was completed as part of a larger research project about mental health and school-based services used by Canadian and immigrant parents. This paper presents results relevant to mental health services, child mental health, school-related barriers, access to resources and information, perceived social support and family demographic information.

#### 2.3.1. Parent and Family Sociodemographic Information

Parents provided information on age, child sex, education, household income, current province and whether they were born in Canada.

#### 2.3.2. Family Mental Health Questionnaire

Parents reported if their child had been referred for or assessed for various NDD/MHs such as ADHD, anxiety, autism spectrum disorder (ASD), speech disorders, intellectual disabilities or other mental health conditions. They also reported on their own mental health difficulties, including anxiety, depression and substance use difficulties.

#### 2.3.3. Accessing Information and Seeking Support Questionnaire

Participants indicated where they access information about support services (e.g., social network, school counsellor, online, healthcare provider) and where they turn for support when their child struggles (e.g., friends, family, school, mental health services). We also used the Multidimensional Scale of Perceived Social Support (MSPSS) to better understand perceived social support. Participants were also asked about communication with school staff and the types of support they received using a 5-point Likert scale (1 = “Strongly disagree” to 5 = “Strongly agree”). The MSPSS is a 12-item self-report measure designed to evaluate an individual’s perception of the level of social support available to them in various domains of their lives [20]. Participants are asked to rate statements, such as “There is a special person who is around when I am in need”, on a 7-point Likert scale (1 = “Very strongly disagree” to 7 = “Very strongly agree”), and higher scores indicate higher perceived social support. The Cronbach’s alpha for this measure with the current sample was 0.96 and the items were strongly correlated with the total score and the 3 individual subscales.

#### 2.3.4. School-Based Barriers

Parents were asked to select from a list whether the following factors were a barrier to their child’s academic success. Items included language skills, social support at school, additional time, extra help, understanding of their culture and access to special education support. Following that, parents were asked to rate the impact of these school-based barriers on a scale of 0 to 100.

### 2.4. Procedure

Eligible participants were contacted via email by AskingCanadians and were provided detailed information about the study, including its purpose, procedures and confidentiality assurances. Interested individuals received a link which directed them to a consent form. After consenting, participants were presented with an eligibility screener to ensure they met the inclusion criteria. Those who were deemed eligible based on the screener were redirected to the anonymous survey. The survey and screener were administered using Qualtrics and took 30–40 min to complete.

### 2.5. Data Analysis Plan

SPSS version 29.1 was used to check survey data for accuracy, normality assumptions and homogeneity of variance. Chi-square tests for categorical (parent education, household income, child sex) and analyses of variance for continuous variables (child age) were assessed for differences between children who were evaluated for NDD/MH (ADHD, ASD, anxiety, behavioural disorders) and those who were not. Chi-squared tests were conducted for binary variables (percentage of parents reporting NDD/MHs assessment referrals and access to treatment, child mental health needs, parent mental health, reported barriers, such as language use, social support, lack of help, educational barriers and limited time) between immigrant and non-immigrant groups. One-way ANOVAs were used for continuous variables (parental support-seeking behaviour and information access, assessing differences in communication with school staff).

## 3. Results

### 3.1. Sample Characteristics

A total of 682 parents completed the full survey as part of the larger online survey study, with 58.6% of the parents identifying as Canadian-born compared to 41.4% of the surveyed parents who reported being born outside Canada. The majority of the respondents identified as being male (54.9%), married or in a common law relationship (85.6%), having at least a Bachelor’s degree (45.3%) and a household income of $100,000 and over (63.1%). The sample recruited was from all provinces in Canada, with the highest representation from Ontario (49.0%), followed by Alberta (17.6%), British Columbia (15.7%) and Quebec (8.4%). The mean age of the child being reported was 13.2 years old (*SD* = 4.10). Of the total sample, 25.1% of the families reported their child being referred for an NDD/MH assessment.

### 3.2. Difference in Sociodemographic Factors

Immigrant families reported significantly higher levels of education compared to Canadian families. They also reported lower English language proficiency, household income and similar financial well-being (See Table A1 for more details). There was no significant difference in child age between the immigrant and non-immigrant parent groups assessed for NDDs/MHs (*F* (1, 600) = 1.04, *p* > 0.05). Among children referred for NDD/MHs assessments whose parents identified as immigrants, 59.2% were male and 40.8% were female, with no significant difference in biological sex distribution (χ^2^ = 0.03, *p* > 0.05). Similar proportions were observed in the non-immigrant parent group, with 50.4% male and 49.6% female (χ^2^ = 0.10, *p* > 0.05). Additionally, there were no significant differences in household income or parent education between the two groups.

### 3.3. Child Needs and Access to Mental Health Services

A Mann–Whitney U Test was conducted to investigate difference in child mental health needs between immigrant and non-immigrant families. Results showed that immigrant parents reported that their children expressed a greater need for mental health services (i.e., mental health professional, school counsellor, online service, community-based support group than children of non-immigrant parents, *U* = 31,073.00, z = 3.36, *p* < 0.001). However, non-immigrant parents from the sample reported accessing mental health services for their children at 1.5 times greater frequency than immigrant parents (Chi-square test, *χ*^2^ = 11.43, *p* < 0.001).

### 3.4. Service Referrals for Child NDD/MHs Assessment

A significant group difference was observed between the percentage of parents who reported that their child had been referred for an NDD/MHs assessment between immigrant (28.7%, *n* = 49) and non-immigrant (71.3%, *n* = 122) groups (χ^2^ = 15.16, *p* < 0.001, Cramer’s V = 0.15) with a significantly higher frequency of Canadian children accessing NDD/MH assessments compared to children of immigrant family background.

### 3.5. Parental Mental Health Challenges

Chi-square tests revealed that parents of children referred for NDD/MHs assessment reported a markedly higher rate of distress associated with mood (40.9% vs. 17.4%; χ^2^ = 39.6, *p* < 0.001, Cramer’s V = 0.24), anxiety (44.4% vs. 16.6%; χ^2^ = 54.9, *p* < 0.001, Cramer’s V = 0.28), sleep disturbances (32.7% vs. 14.7%; χ^2^ = 26.9, *p* < 0.001, Cramer’s V = 0.20), substance use (8.8% vs. 3.9%; χ^2^ = 6.21, *p* < 0.01, Cramer’s V = 0.10), trauma-related issues (13.5% vs. 4.3%; χ^2^ = 17.4, *p* < 0.001, Cramer’s V = 0.16) and disordered eating (8.8% vs. 3.7%; χ^2^ = 6.91, *p* < 0.01, Cramer’s V = 0.10). This pattern was also observed when looking at the subset of immigrant parents only with higher rates of distress associated with mood (40.8% vs. 15.5%; χ^2^ = 16.4, *p* < 0.001, Cramer’s V = 0.24), anxiety (36.7% vs. 13.7%; χ^2^ = 14.7, *p* < 0.001, Cramer’s V = 0.23) and sleep disturbances (42.9% vs. 15.9%; χ^2^ = 18.0, *p* < 0.001, Cramer’s V = 0.25). The results associated with substance use, trauma-related issues and disordered eating were not reported due to the significantly smaller sample size (See Table A2 for detailed comparative data).

### 3.6. Seeking Support and Information Access

During periods of difficulty, parents whose children have reported NDD/MH concerns predominantly sought support from family members (50.3%), their child’s school (28.7%) and mental health services (29.8%) regarding social-emotional challenges in their children. While searching for information about available support services, parents primarily relied on healthcare providers (51.1%) and online sources (38.9%). Notably, there were no statistically significant differences between immigrant and Canadian parents of children assessed for NDD/MHs and those not assessed regarding their perceptions of the academic support and resources provided by schools or teachers (*F* = 1.0, *p* = 0.34). Similarly, no significant differences were observed between the two groups concerning their experiences with communication with schools or teachers (*F* = 0.22, *p* = 0.64).

### 3.7. Perceived Social Support

The results from the MANOVA reported a significant interaction effect, with Canadian-born parents of children with NDD/MH reporting significantly less perceived social support compared to immigrant parents of children with NDD/MH, *F* (3, 530) = 3.28, *p* = 0.02, Partial Eta squared = 0.02. Univariately, Canadian-born parents reported receiving less support from significant others, *F* (1, 535) = 4.08, *p* = 0.04, partial Eta squared = 0.01 as well as from friends, *F* (1, 535) = 5.03, *p* = 0.03, partial Eta squared = 0.01, than immigrant participants. No significant difference was observed for the family subscale, *F* (1, 535) = 0.50, *p* > 0.05.

### 3.8. School Related Barriers

Parents of children presenting with NDD/MH concerns overall reported significantly higher impact of barriers on their child’s education compared to parents who did not present with NDD/MH concerns (*F* (1, 227) = 8.33, *p* < 0.01, partial Eta squared = 0.04). These reported school-based barriers did not vary between immigrant and non-immigrant families, *F* (1, 227) = 0.05, *p* > 0.05. However, there was an interaction effect, with Canadian parents of children with NDD/MH concerns reporting more barriers than immigrant parents (*F* (1, 227) = 4.72, *p* < 0.05, partial Eta squared = 0.02). Reported school-based barriers for immigrant families compared to non-immigrants included language barriers (14.3% versus 8.2%), less social support at school (18.4% versus 23.8%), less additional help for their children (26.5% versus 18.0%) and limited access to special education resources (14.3% versus 15.6.%), compared to parents whose children were not referred for NDDs related services

## 4. Discussion

The present study aimed to explore the differences in MH challenges reported by immigrant parents for their children, NDD and MH service referrals, parental mental health challenges, support-seeking behaviours between immigrant and non-immigrant families in Canada and school-related barriers. Specifically, we used an online survey to compare differences between immigrant and non-immigrant families.

Results from the study found that immigrant parents reported a greater need for mental health services for their children than non-immigrant parents, while non-immigrant parents reported accessing mental health services for their children at a higher rate than immigrant parents. It is possible that children may express the perceived need for mental health support, but their challenges are inadequately addressed by their families, considering that immigrant families utilize support 1.5 times less than their non-immigrant counterparts. When specifically looking at specialized service referrals, such as a diagnostic assessment for NDD/MH concerns, we found that immigrant parents reported lower rates of NDD/MH assessment referrals. Cultural and language barriers may prevent immigrant families from recognizing NDD/MHs symptoms or seeking appropriate assessments. Additionally, a lack of awareness or understanding of the Canadian healthcare system and available mental health services could contribute to these lower referral rates. These findings align with previous research indicating that immigrant families often face barriers in accessing mental health services due to cultural beliefs, language barriers and lack of familiarity with the healthcare system [14,19].

Parents of children referred for NDDs assessment reported a higher prevalence of mental health issues, including mood disorders, anxiety disorders and sleep disturbances, compared to parents of children not referred for NDD/MHs assessment. These rates did not differ for immigrants versus non-immigrants. This association suggests that parental mental health is closely linked to the mental health of their children. These findings underscore the importance of providing comprehensive support to families dealing with NDD/MHs, addressing both the child’s and parents’ mental health needs.

In terms of seeking support for their child’s mental health, parents of children with NDD/MH concerns primarily sought support from family members, their child’s school and mental health services during difficult times. Healthcare providers and online sources were the main avenues for accessing information about support services. Additionally, there were no differences between parents of children assessed for NDD/MHs and those not assessed regarding their perceptions of the academic support and resources provided by schools or teachers. However, when investigating perceived social support, Canadian-born parents of children with NDD/MH concerns reported significantly less perceived social support compared to immigrant parents of children with NDD/MH. Furthermore, Canadian families of children with NDD/MH concerns reported more school-based barriers. While these results are in contrast to what was expected, the results could suggest that immigrant families of children with NDD/MH feel more supported in Canada due to inequity in service and healthcare access in their country of origin [21]. Access to mental health and NDD support in non-western countries is generally limited and has been a long-withstanding area of concern [22]. When immigrant families find themselves in their new environment, they are more appreciative of the availability of resources. This might not be the same experience for Canadian families. These findings further highlight the need for improved communication and support systems within schools to better serve all families dealing with NDD/MHs.

### 4.1. Implications for Practice and Policy

To improve access to mental health services for immigrant families, it is crucial to address cultural and language barriers through the development of culturally sensitive and accessible resources. Providing information in multiple languages and involving community leaders and organizations in outreach efforts could help bridge these gaps. Additionally, enhancing parental education and awareness about NDD/MHs and available support services could empower families to seek help earlier.

Policymakers should consider the unique challenges faced by immigrant families and work towards ensuring equitable access to mental health services, reducing administrative barriers and providing additional support to families navigating the healthcare and educational systems. By addressing these issues, it is possible to improve the mental health and educational outcomes of children with NDD/MHs and their families.

### 4.2. Strengths and Limitations

The findings of the present study should be considered in the context of several limitations, such as potential sampling bias due to recruitment methods (e.g., online survey conducted through a crowdsourcing website), small sample size, a cross-sectional design limiting causal inferences, reliance on self-report measures which are susceptible to biases and not accounting for other potential confounding factors such as acculturation effects. This study recruited participants through a crowdsourcing platform in order to mitigate issues such as responses from bots and data farming that are common in social media recruitment. While crowdsourcing platforms may not be commonly used by many immigrant families and likely represent a subset of immigrant families’ experience, we were able to offer an understanding of what immigrant experiences could be like. These limitations warrant caution in interpreting and applying the findings to a broader population. Future research should address these issues by recruiting participants through more targeted community agencies to enhance the study’s validity. Given that immigrant families are diverse with different experiences that cannot be captured through surveys, future research with more qualitative interviews is required to identify barriers and positive factors in immigrant families. Future studies could also consider the impact of culture and cultural norms as they pertain to mental health and service use.

## 5. Conclusions

The present study highlights the significant disparities in MH needs, NDD/MHs assessment and service referrals and parental mental health challenges between immigrant and non-immigrant families. Future research and policy efforts should focus on developing culturally sensitive, accessible and comprehensive support systems to meet the unique needs of immigrant families. The present study underscores the significant disparities in NDD assessment referrals and parental mental health challenges between immigrant and non-immigrant families in Canada. Parents, regardless of their child’s NDD assessment status, face similar difficulties in seeking support and navigating the educational system. There is a need for improved communication and support to better serve families dealing with NDDs, highlighting the need for tailored support services to address these challenges effectively. By addressing these disparities and enhancing access to mental health services and educational support, it is possible to improve the well-being of children with NDDs and their families, ultimately contributing to better mental health and educational outcomes.

## Data Availability

De-identified individual-level self-report data and SPSS syntax that underlie manuscript findings will be made available upon reasonable request. Requests for data should be directed jointly to the study senior author (T.H.) at tasmia.hai@mcgill.ca. All requests should detail the reason for the request and describe how the data will be used.

## Appendix A

**Table A1:** Demographic and social characteristics of the survey sample.

		Full Sample	Immigrants	Non-Immigrants	*p* Value ^a^
		*n* = 682	*n* = 282	*n* = 400	
Child age		13.2 (4.1)	13.0 (4.1)	13.4 (4.0)	0.27
Participant age		31.8 (7.4)	31.6 (7.4)	31.9 (7.3)	0.8
Parent Sex (% females)		46.1	40.8	49.6	0.03
Number of Children		2.0 (1.0)	1.9 (0.98)	2.0 (1.0)	0.4
Financial Wellbeing ^b^		2.97 (0.94)	2.94 (0.9)	2.99 (0.96)	0.4
English skills ^d^		18.45 (2.68)	17.80 (2.87)	18.89 (2.44)	<0.001 *
Household Income ^e^	$0–$39,000	6.1	6.8	5.7	0.04
	$40,000–$69,999	12.2	14.3	10.8	
	$70,000–$99,999	18.3	23.7	14.7	
	$100,000–$124,999	15.9	16.5	15.5	
	$125,000–$149,999	12.7	9.8	14.7	
	$150,000–$199,999	13.6	11.7	14.9	
	$200,000+	15.0	11.7	17.3	
Educational Levels ^e^					<0.001 *
	Partial/full high school	23.2	12.4	30.7	
	College/Bachelors Degree	52.3	55.6	50.0	
	Graduate Degree	23.9	30.8	19.1	
Province of Residence ^e^					
	Alberta	17.6	15.9	18.8	
	Ontario	49.0	55.1	44.8	
	British Columbia	15.7	14.5	16.5	
	Manitoba	2.5	1.8	3	
	Saskatchewan	2.1	2.2	2	
	Quebec	8.4	9.1	8	
	New Brunswick	1.3	0.7	1.8	
	Newfoundland and Labrador	1.0	0.4	1.5	
	Nova Scotia	2.2	0.4	3.5	
	Prince Edward Island	0.1	0	0.3	
Years in Canada ^e^	0–5 years		9.5		
	6–10 years		16.4		
	11–15 years		17.8		
	16–20 years		14.5		
	21–25 years		14.2		
	26+ years		27.6		
Ethnicity ^e^					
	Indigenous	1.6	0.5	2.2	
	Latin American	2.8	6.3	1.4	
	East Asian	18.0	23.8	15.7	
	Indo-Caribbean	0.1	0.5	0.0	
	Black Caribbean	1.7	2.1	1.6	
	South Asian	9.7	28.0	2.8	
	Middle Eastern	3.8	2.1	4.4	
	Southeast Asian	4.4	2.1	5.2	
	White Canadian/American	39.8	7.4	51.7	
	White European	13.7	15.9	13.3	
	Black Canadian/African American	0.4	0.5	0.4	
	Black African	2.3	7.9	0.2	
	Other	1.6	2.6	1.2	

^a^ *p*-values represent the results of a One-way ANOVA (age, household income) or Chi-square analysis (education) between immigrant and non-immigrant groups. ^b^ Financial wellbeing represents Likert scale data from 1–5, with 1 being very well off, and 5 being not well off. ^d^ English skills are calculated as a mean total score of four Likert-scale questions (conversation, writing, reading and media literacy), verified through reliability analysis. ^e^ Data are shown as n (%) of participants from each category. * Significance after Bonferroni correction (α = 0.008).

**Table A2:** Self-reported mental health challenges of immigrant parents whose children had and had not been referred for an NDD/MHs assessment.

	Immigrants		
	Received NDD/MH Service (*n* = 49)	Did Not Receive NDD/MH Service (*n* = 233)	*p* Value ^a^
Mood related distress	20 (40.8%)	36 (15.5%)	<0.001
Anxiety related distress	18 (36.7%)	32 (13.7%)	<0.001
Sleep Challenges	21 (42.9%)	37 (15.9%)	<0.001
Trauma-related distress	7 (14.3%)	12 (5.2%)	0.02
Others (including substance use, eating challenges)	3 (6.1)	22 (9.44)	Ns

^a^ *p*-values represent the results of a Chi-square analysis, and were considered for statistical significance at *p* = 0.01. Note: parent mental health data was gathered through a checklist.
